# Osmotic laxatives do not alter dabigatran plasma concentration in healthy volunteers – a randomized, controlled, cross-over trial

**DOI:** 10.3389/fphar.2025.1579014

**Published:** 2025-05-12

**Authors:** Matthias Weiss-Tessbach, Al Medina Dizdarevic, Alexander Kupis, Thorsten Bischof, Christa Firbas, Peter Quehenberger, Ulla Derhaschnig, Max Frimmel, Bernd Jilma, Christian Schoergenhofer

**Affiliations:** ^1^ Department of Clinical Pharmacology, Medical University of Vienna, Vienna, Vienna, Austria; ^2^ Department of Laboratory Medicine, Medical University of Vienna, Vienna, Vienna, Austria

**Keywords:** dabigatran, laxatives, pharmacokinetics, drug-drug interactions, bioavailability, anticoagulation therapy

## Abstract

**Background:**

Laxatives are among the most commonly used pharmacological agents worldwide. Available data indicate a significant potential for clinically relevant drug-drug interactions. We hypothesized that osmotic laxatives may reduce the oral bioavailability of the direct oral anticoagulant dabigatran and thereby its anticoagulant effects.

**Methods:**

In the first part of this single-centre, randomized, double-blind, crossover trial, 24 healthy volunteers received 150 mg dabigatran with placebo (10 g glucose) or 20 g lactulose. In the second, open label part, eight of these 24 healthy volunteers were randomly assigned to receive dabigatran with either 27.6 g macrogol, 30 g flaxseeds, or to receive 20 g lactulose 4-h after dabigatran intake. We measured dabigatran plasma concentrations using an ecarin-based chromogenic assay and calculated the pharmacokinetic parameters. Statistical analysis was performed using a linear mixed-effects model on log-transformed AUC values.

**Results:**

The main pharmacokinetic parameters AUC, C_max_, T_max_, or t_1/2_ did not differ significantly between most treatment periods. A reduction in AUC was observed with flaxseed compared to placebo. Dabigatran’s pharmacokinetics remained unaffected by concomitant intake of lactulose or macrogol. There was a high inter- and intra-individual variability in the pharmacokinetics of dabigatran.

**Conclusion:**

In this study osmotic laxatives such as lactulose, macrogol or flaxseeds did not affect the pharmacokinetics of dabigatran in healthy individuals. These findings support the safe concurrent use of dabigatran with osmotic laxatives.

## Introduction

Laxatives are among the most widely utilized pharmacological agents globally. Constipation is a functional bowel disorder characterized by persistently difficult, infrequent, or incomplete defecation. The prevalence of constipation in adults ranges widely, from 2.6% to 26.9%, but increases substantially in individuals over 65 years of age, with rates between 24% and 50% (Talley et al., 1992; Choung et al., 2007; Schmidt and de Gouveia Santos, 2014). In nursing home populations, constipation is even more pervasive, with up to 74% of residents using laxatives at least once daily (Harari et al., 1994; Roerig et al., 2010).

Despite their widespread use, limited data exist regarding the potential of laxatives to cause drug-drug interactions (DDI). Available evidence indicates that laxatives can significantly reduce the absorption of concurrently administered oral drugs. For example, macrogol has been reported to reduce the maximum concentration of digoxin by 40% and the area under the concentration-time curve (AUC) by 30% (Ragueneau et al., 1999). Similarly, an osmotic load containing lactulose, a non-absorbable osmotic agent, has demonstrated a substantial impact on the pharmacokinetics (PK) of atenolol, decreasing its maximum concentration by 80%–90% and its AUC by 70%–90% in healthy volunteers (Riley S. et al., 1992). This study reported similar results for furosemide and hydrochlorothiazide intake, whereas the absorption of acetylsalicylic acid remained unaffected. Additionally, some case reports indicate an impaired absorption of levothyroxine. One possible explanation may be that in contrast to acetylsalicylic acid all other discussed drugs are poorly permeable and the biopharmaceutics classification system (BCS) classifies them as class III (good solubility, poor permeability) or IV drugs (poor solubility, poor permeability). Acetylsalicylic acid is a BCS class I drug, characterized by good solubility and good permeability.

Another possible mechanism for these interactions involves the laxative-induced increased influx of water into the intestinal lumen diluting intestinal contents. Osmotic laxatives bind this water and all dissolved substances intra-luminally, ultimately impairing the absorption of passively absorbed drugs. While this hypothesis requires further investigation, the potential implications for patients on critical therapies, for instance direct oral anticoagulants (DOACs), merit investigation. Dabigatran, a direct thrombin inhibitor, is a BCS class II drug (poor solubility, good permeability) that is characterized by a poor oral bioavailability of only 6%, which may possibly predispose for such a DDI (Blech et al., 2008). Dabigatran exhibits a direct concentration-effect relationship, and reduced absorption may lead to an increased risk of thromboembolic events, such as stroke and pulmonary embolism (Steffel et al., 2021). Given the frequent use of both laxatives and dabigatran, investigating a potential DDI is essential to reduce risks and improve patient safety.

This study investigated the influence of commonly used osmotic laxatives - including lactulose, macrogol, and natural alternatives such as flax seeds - on the PK of dabigatran. Furthermore, we investigated the PK of dabigatran, when a time gap of 4 h between drug and laxative intake is maintained.

## Material and methods

### Ethics

This trial complied with the principles set forth in the International Conference on Harmonization–Good Clinical Practice (ICH-GCP) guidelines and the Declaration of Helsinki. The study was registered at the EudraCT database (number 2018-004697-10) and received approval from the local Ethics Committee of the Medical University of Vienna (number 2254/2018) and the Austrian Agency of Health and Food Safety. All participants gave their written informed consent prior to any study related intervention.

### Study design

**FIGURE 1:**
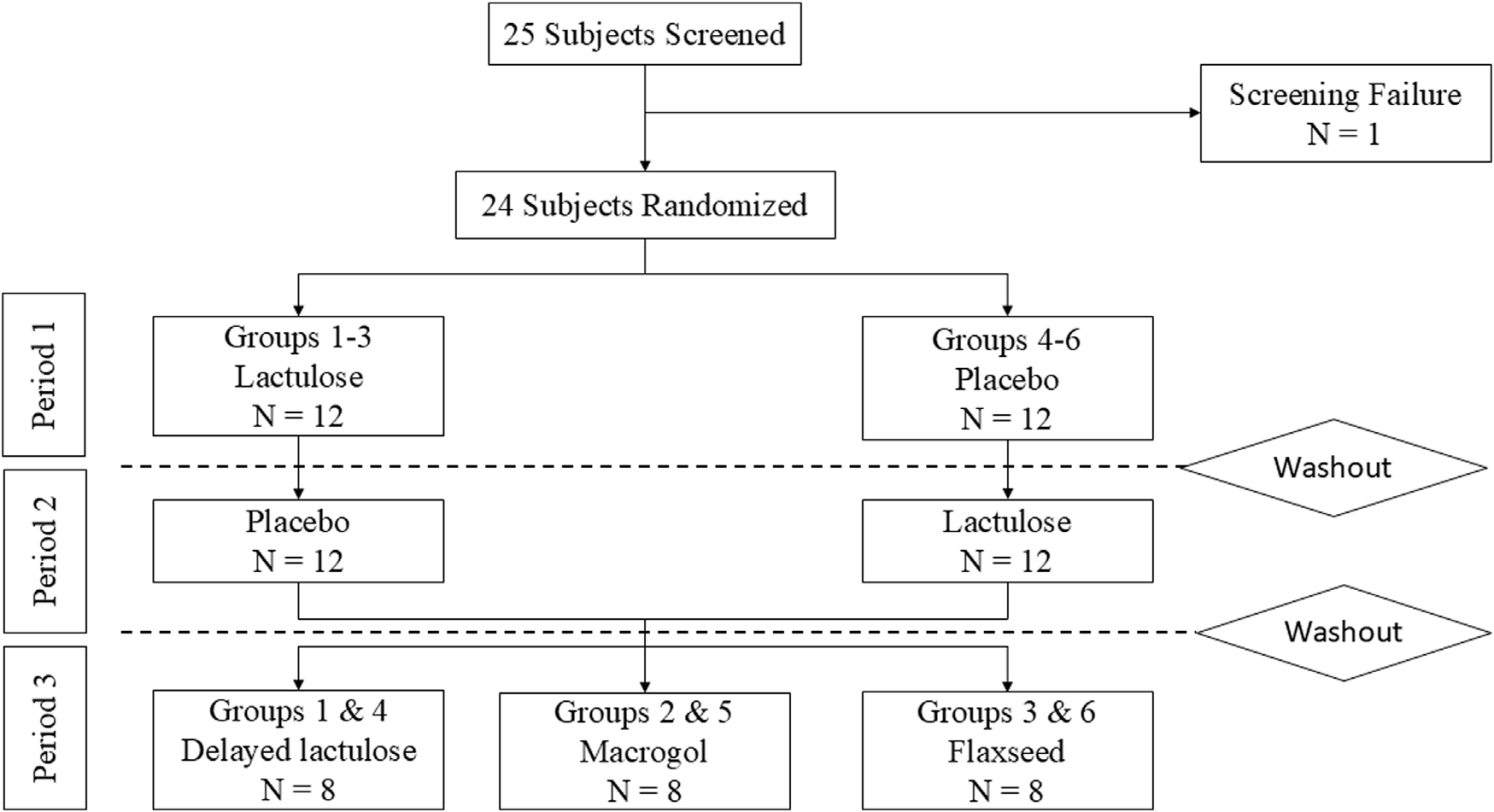
Subject flowchart. Dabigatran was given in all periods. The washout period was at least 1 week after the last dose of Dabigatran.

A detailed description of the study design and inclusion/exclusion criteria can be found in the Supplementary Table S1. Participants fasted for 8 h prior to drug administration. 2 h post-dosing subjects received a standardized breakfast followed by a standardized lunch 6 h post-dosing. Subjects were allowed to drink tap water *ad libitum* throughout the study. Study nurses assigned sequential numbers to participants. Unblinded pharmacists randomized healthy volunteers into six treatment groups by entering these numbers into a web-based randomization software with permuted blocks of variable size generated by the pharmacist before study initiation (Figure 1). In the first period, groups 1-3 received a placebo with the study drug, while groups 4-6 received lactulose with the study drug. After a washout period of at least 7 days participants received the respective other treatment. This part of the study was a randomized, double-blind, crossover trial. Study staff and participants were blinded to the treatment, which was indistinguishable in taste, smell or physical appearance. The second part was an open label study that commenced following a second washout period of 7 days. Groups 1 and 4 received lactulose 4 h after the study drug. Groups 2 and 5 received macrogol, while groups 3 and 6 received flaxseeds with the study drug simultaneously.

### Substances and materials

All study medications were prepared by an unblinded pharmacist. Dabigatran etexilate 150 mg capsules (Pradaxa^®^, Boehringer Ingelheim, Germany) were administered orally. Osmotic laxatives, including lactulose 20 g (Laevolac^®^, Fresenius Kabi, Austria), macrogol 27.6 g (Movicol^®^, Laboratoires Macors, France), and rough-ground flaxseeds 30 g (allowed to swell for ≥10 min), as well as the placebo (10 g glucose), were dissolved in 250 mL water and administered orally. To match the sweetness of lactulose and minimize the risk of unblinding, glucose was used as the placebo.

### Outcome and assessments

The primary objective of this study was to investigate the effect of lactulose on the PK of dabigatran with the primary endpoint being the area under the concentration-time curve (AUC). Secondary endpoints included the maximum plasma concentration (C_max_), the time to reach the C_max_ (T_max_) and the terminal elimination half-life (t_1/2_) of dabigatran. Likewise, we investigated the impact of other osmotic laxatives on the PKs of dabigatran. Finally, we investigated whether a 4-h time-gap (delayed lactulose) had any relevant impact on the PKs of the study drug. Blood samples were collected at baseline and at 15 min, 30 min, 1, 2, 4, 6, 8, and 24 h following the intake of the study drug. Dabigatran plasma concentrations were quantified using a validated Ecarin-based chromogenic assay (ECA II, Diagnostica Stago, France) performed at the Department of Laboratory Medicine, Medical University of Vienna, in accordance with the manufacturer’s instructions. This assay is routinely used and approved for dabigatran monitoring in clinical practice. Blood samples were collected into 3.2% sodium citrate tubes (Vacuette, Greiner Bio-One, Austria), centrifuged at 2000 × g for 15 min, and plasma was either immediately analyzed or stored at −80°C until measurement. Additionally, thrombin time (STA-Thrombin, Diagnostica Stago, France) was assessed at baseline and 2, 4 and 24 h following study drug intake.

### Statistical methods

Pharmacokinetics were calculated using Phoenix WinNonLin (Certara, NJ, United States). Normality of data distribution was evaluated with the Shapiro-Wilk test. Differences in outcome parameters between treatment groups were analyzed using a linear mixed-effects model with treatment as a fixed effect and subject as a random effect. The model was fitted to log_10_-transformed AUC values using the lmer function from the lmerTest package (version 3.1-3) in R (version 4.4.3), with restricted maximum likelihood estimation disabled (REML = FALSE). Two-sided p-values < 0.05 were considered statistically significant. Data visualization was performed in GraphPad Prism version 10.4.1 (GraphPad Software, Boston, United States). Details of the sample size calculation are provided in the Supplementary Material.

## Results

### Participants

This single-centre, double-blind, crossover trial, enrolled 24 healthy volunteers (15 females, 9 males) between March 2024 and June 2024. One participant failed screening due to elevated creatinine levels. All 24 participants completed all three study periods as of August 2024 (Figure 1).

The mean age was 36 (SEM ± 7.4) years, and the mean BMI was 25 kg/m^2^ (SEM ± 4.9) (Supplementary Table S2). There were no serious adverse events, and no adverse events related to the study drugs during the study (Supplementary Table S3). Additionally, there were no clinically significant abnormalities in laboratory parameters at screening, prior to dosing, or 24 h post-dosing (Supplementary Table S4).

**FIGURE 2:**
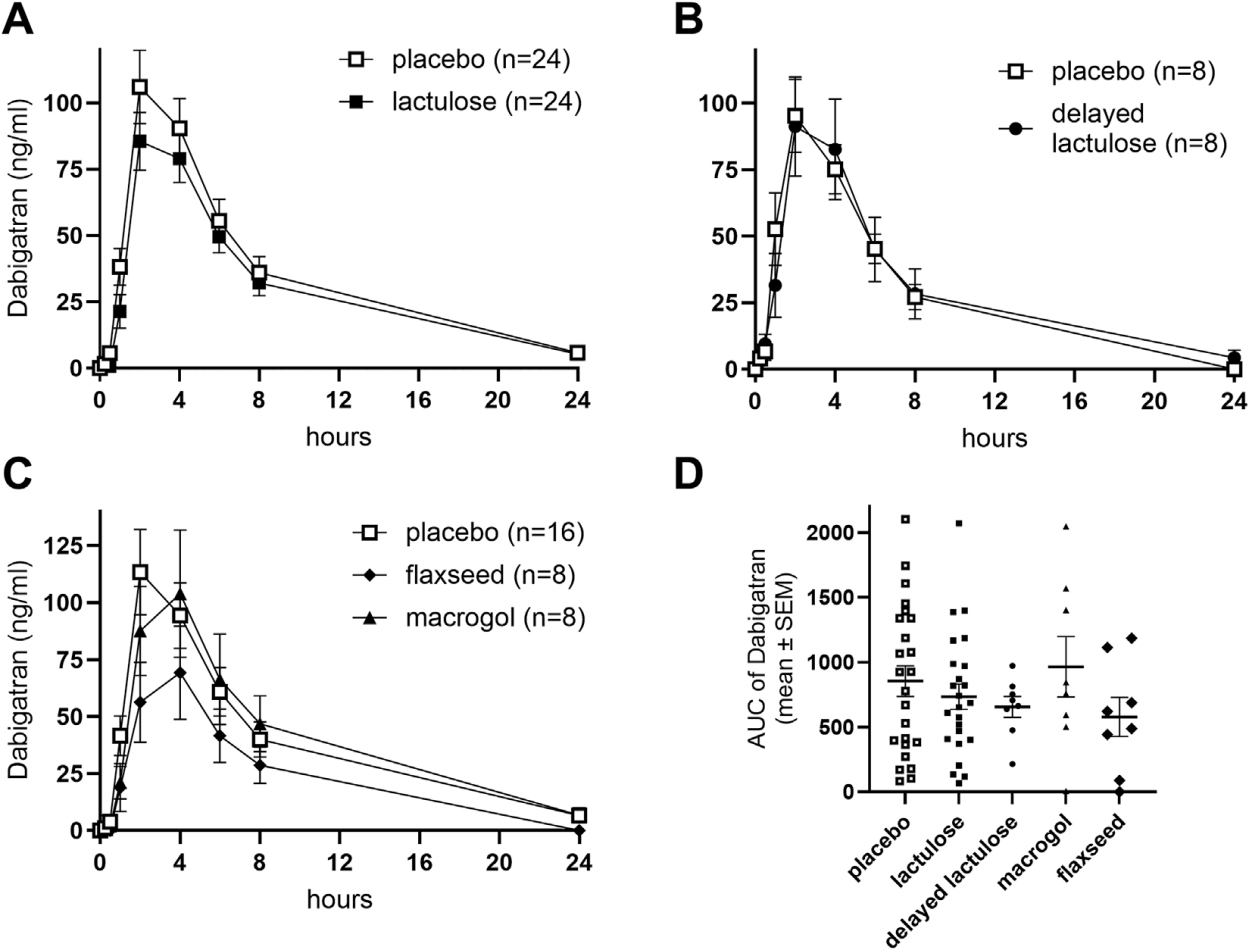
Effects of different laxatives and timing of drug intake on the pharmacokinetics of dabigatran. Concentration-time profiles are shown for placebo vs. lactulose **(A)**, placebo vs. delayed lactulose group **(B)**, placebo vs. macrogol, and flaxseeds **(C)**. AUC (ng/ml*h) comparisons across all groups and periods are shown in **(D)**. Placebo data in each panel were derived from the respective participants’ placebo period to allow within-subject comparisons across study arms. SEM: Standard error of the mean. Delayed lactulose: 4-h interval between lactulose and dabigatran intake.

The AUC of dabigatran did not differ significantly between the placebo and lactulose periods (p = 0.726, 95% CI [–0.246, 0.171]; Figures 2A, D), the delayed lactulose (*p* = 0.782, 95% CI [–0.272, 0.362]; Figures 2B, D), or the macrogol period (p = 0.241, 95% CI [–0.511, 0.126]). However, AUC was significantly lower during the flaxseed period compared to placebo (p = 0.043, 95% CI [–0.594, −0.017]; Figures 2C, D).

Secondary endpoints including C_max_ (Supplementary Figure S1), T_max_ and terminal elimination half-life did not differ significantly between study periods (Table 1).

**TABLE 1:** Pharmacokinetic parameters of dabigatran across different study groups.

Parameter	Placebo	Lactulose	Delayed lactulose	Flaxseed	Macrogol
AUC (ng/ml*h)	854 ± 118	735 ± 97	655 ± 81	578 ± 151	965 ± 234
p vs. placebo^#^	—	0.726	0.782	0.043	0.241
95% CI (log_10_ AUC)^#^	—	−0.246, 0.171	−0.272, 0.362	−0.594, −0.017	−0.511, 0.126
Cmax (ng/mL)	114 ± 14	96 ± 11	101 ± 10	77 ± 18	115 ± 24
Tmax (h)	2.5 ± 0.2	2.7 ± 0.2	2.4 ± 0.4	2.9 ± 0.4	2.5 ± 0.5
t½ (h)	3.9 ± 0.8	4.5 ± 0.9			

Values are presented as mean ± SEM (standard error of the mean). AUC: area under the plasma concentration–time curve; C_max_: maximum plasma concentration; T_max_: time to reach C_max_; t½: elimination half-life.

^#^ p-values and 95% confidence intervals (CI) for AUC were derived from a linear mixed-effects model on log_10_-transformed AUC values, using placebo as the reference.

Thrombin time was evaluated as a secondary pharmacodynamic measure. No significant differences were found in the AUC for thrombin time (mean ± SEM) between the placebo (1,591 ± 72.5) and lactulose groups (1,553 ± 85.5), as well as for placebo vs. other laxatives (Supplementary Figure S2).

## Discussion

This study investigated the impact of commonly used laxatives lactulose, macrogol, and flaxseeds on the PK of dabigatran in healthy volunteers. There were no significant differences in the PK of dabigatran across treatment groups and periods, except for a borderline difference in the flaxseed period with a limited effect size, indicating the absence of a clinically relevant DDI. Thus, our findings may support the safe co-administration of dabigatran and these laxatives in clinical practice.

These results differ from previous findings suggesting that osmotic laxatives may reduce the bioavailability of certain concomitantly administered drugs. Riley S. et al. (1992) showed that hyperosmolar, multi-component solutions reduced atenolol’s AUC by 70%–90%, with similar results shown for hydrochlorothiazide and furosemide (Riley S. et al., 1992). Furthermore, such drug-drug interactions were reported for digoxin and levothyroxine (Wang et al., 1990; Mersebach et al., 1999). Of note, Riley et al. found no impact of osmotic solutions on the PK of acetylsalicylic acid. One possible explanation is that the potential for DDIs of osmotically active laxatives is limited to poorly permeable drugs (BCS classes III and IV). The underlying hypothesis is that the absorption of poorly permeable drugs is time-dependent and one major determinant of absorption therefore is the gastrointestinal transit time, which is shortened by osmotic laxatives (Chen et al., 2013; Vinarov et al., 2021). Chen et al. reviewed the influence of various osmotically active excipients on the bioavailability of BCS class III drugs (Chen et al., 2013). They concluded that there is a linear relationship between the osmotic potential, gastrointestinal motility and drug absorption for BCS class III drugs, while such a relationship was not evident for BCS class I drugs. Riley et al. also reported significantly shorter gastro-coecal transit times after treatment with the osmotic solutions (Riley S. et al., 1992). The same group also studied how metoclopramide and codeine, as gastrointestinal transit modifiers, influence the PK of atenolol (BCS class III) and furosemide (BCS class IV). Surprisingly, they did not find an association between absorption and gastrointestinal transit time, which was shorter after metoclopramide treatment and longer after codeine treatment (Riley S. A. et al., 1992). These findings raise the question, whether the drug-drug interaction potential of osmotic laxatives is solely explainable by gastro-intestinal transit time. Vinarov et al. emphasized that although the mechanism seems obvious, there are only few studies that have actually investigated the relationship between gastrointestinal transit times and drug absorption (Vinarov et al., 2021). With regard to our study, we did not measure gastro-coecal transit times, which is an obvious limitation.

Dabigatran is classified as BCS Class II, characterized by a low solubility and a high permeability, which may possibly explain the lack of effect in our study (U.S. Food and Drug Administration, 2011). However, dabigatran is only absorbed to a small extent with an oral bioavailability of approximately 6.5% making it an interesting target (Stangier et al., 2005). Dabigatran is a substrate of P-glycoprotein (P-gp) and hence, several clinically relevant drug-drug interactions have been reported based on this mechanism (Blair and Keating, 2017). Importantly, there are no data suggesting that the laxatives used in this study interact with P-gp. Furthermore, dabigatran is poorly soluble at a pH > 4 and a mild pharmacokinetic DDI has been reported with pantoprazole and esomeprazole, which, however, is of questionable clinical relevance (Ferri et al., 2022). In the phase III trial, concomitant intake of proton pump inhibitors did not impact on clinical outcomes (Connolly et al., 2009). Lactulose is metabolized by colonic bacteria and short fatty acids are formed that are (i) osmotically active and (ii) acidify the colon. Importantly, lactulose is not considered to affect the pH in the upper gastrointestinal tract. The same holds true for macrogol and for flaxseed. Concomitant intake of food delays the Tmax by approximately 2 h, although, it has no effect on oral bioavailability (Blair and Keating, 2017; Grześk et al., 2021). To reduce potential variability, all subjects in our study received dabigatran after an overnight fast.

However, apart from BCS classes, there are some important methodological differences between our study and the study by Riley S. et al. (1992) They used hyper-osmolar, multicomponent solutions containing mannitol or glucose and electrolytes alongside lactulose, whereas we only used lactulose, macrogol or flaxseeds. The one-component solutions used in our study were hypo-osmolar (lactulose diluted in water) or almost normo-osmolar (macrogol diluted in water), while flaxseeds do not really create a solution in the classical sense. Furthermore, the healthy volunteers in our study were allowed to drink water *ad libitum*, while in Riley’s study this was only allowed 4 h after drug intake (corresponding to 2 p.m.). Possibly, the hyper-osmolar nature of the solution and the lack of fluid intake forced water to move intraluminally until the osmolarity was equilibrated, whereas only negligible amounts of water were actually absorbed. Dissolved drugs were therefore also bound intraluminally, while the unlimited drinking quantity in our study allowed the drugs to distribute and to be absorbed, together with water. However, this explanation requires confirmation and at this point is only hypothetical. When comparing the methodology of the two studies, we believe that our study is clinically more relevant, because, first of all, the osmotic solutions used by Riley et al. are not commercially available. Furthermore, constipated patients are required to ensure adequate hydration and not drinking any water for 4 h after intake of laxatives is not recommended and also unlikely in real world situations (Fresenius Kabi Austria GmbH, 2022). Finally, diluting osmotic laxatives in water may improve tolerability. However, these differences also highlight the complexity of drug-laxative interactions and the need for systematic studies across different BCS classifications with a uniform study design to confirm that this drug-drug interaction is only relevant for poorly permeable drugs.

While AUC values during flaxseed treatment were significantly lower compared to placebo, this finding was borderline and should be interpreted with caution given the multiple comparisons performed. Larger, adequately powered studies are needed to confirm whether this reflects a true pharmacokinetic effect. In addition, the observed difference in the AUC is unlikely to be of clinical relevance. Proton pump inhibitors reduced the AUC of dabigatran to a similar extent, while this effect did not influence clinical outcomes (Connolly et al., 2009) Furthermore, the observed pharmacokinetic effects did not translate into altered thrombin time, a pharmacodynamic biomarker of dabigatran.

The study was designed as a crossover study, aiming to reduce the impact of interindividual variability between treatment periods. Dabigatran plasma levels were below the lower limit of detection at the start of each treatment period, confirming sufficient washout between doses. However, despite these comparable starting points, there was a substantial inter- and intra-individual variability in dabigatran PKs (Supplementary Figure S3), which may have limited the study’s ability to detect significant treatment effects. The coefficient of variation was 66% in the placebo group and 63% in the lactulose group, which exceeds previously reported inter-individual estimates of 27%–43% (Stangier et al., 2007). General factors such as gastrointestinal fluid secretion, diet, gastrointestinal motility and fasting patterns may contribute to variability in drug absorption (Vinarov et al., 2021; Staniszewska et al., 2024). Interestingly, Staniszewska et al. demonstrated in an *in vitro model* a significant impact of gastrointestinal motility on the dissolution of dabigatran capsules (Staniszewska et al., 2024). In addition, Bermejo et al. observed a remarkable variability of ibuprofen plasma concentrations in fasting healthy volunteers and reported that ∼40% of this variability may be explained by the timing of the migrating motor complex III that is responsible for gastrointestinal emptying (Bermejo et al., 2018). The latter may obviously be affected by food intake.

A key limitation of this study is the absence of multiple-dosing regimens, leaving the effects of laxatives on steady-state PK unclear. While previous data suggest that dabigatran’s PK are consistent at steady state, the present findings cannot rule out the possibility of altered plasma concentrations with prolonged laxative use (Stangier et al., 2007). Future studies involving multiple-dosing regimens in a real-world population with comorbidities such as gastrointestinal disorders or chronic constipation are warranted to address this gap. In addition, we did not draw blood 12 h after drug intake for organizational issues and hence, we did not quantify “trough” concentrations.

In conclusion, this study demonstrates that lactulose, macrogol, and flaxseeds do not significantly affect the pharmacokinetics of dabigatran in healthy volunteers.

## Data Availability

The raw data supporting the conclusions of this article will be made available by the authors, without undue reservation.
