# Supplementary material for: Osmotic laxatives do not alter dabigatran plasma concentration in healthy volunteers – a randomized, controlled, cross-over trial
Source: Front Pharmacol. 2025 May 12;16:1579014. doi: 10.3389/fphar.2025.1579014 (PMC12104081; doi:10.3389/fphar.2025.1579014)
Supplement: Supplementary file 1 [file DataSheet1.pdf]

Supplement to:

# Osmotic laxatives do not alter dabigatran plasma concentration in healthy volunteers – a randomized, controlled, cross-over trial

Short Title: Laxatives and dabigatran in healthy volunteers

Matthias Weiss-Tessbach[1], Al Medina Dizdarevic[1], Alexander Kupis[1], Thorsten Bischof[1], Christa Firbas[1], Peter Quehenberger[2], Ulla Derhaschnig[1], Max Frimmel[1], Bernd Jilma[1], Christian Schoergenhofer[1*]

[1] Department of Clinical Pharmacology, Medical University of Vienna, Vienna, Austria
[2] Department of  Laboratory Medicine, Medical University of Vienna, Vienna, Austria

***Corresponding author:**
Christian Schoergenhofer
Department of Clinical Pharmacology
Medical University Vienna
Waehringer Guertel 18-20, 1090 Vienna, Austria
Tel.: +43 1 40400 29850
Fax.: +43 40400 29980
Email: christian.schörgenhofer@meduniwien.ac.at

**Study design:**

**Supplementary Table 1**: Visit and Assessment Schedule

| PERIODS | Name | SCREENING | TREATMENT | | | | FOLLOW-UP |
|---|---|---|---|---|---|---|---|
| VISITS | Number | 1 | 2 | 3 | 4 | 5 | 6 |
| | Name | Screening[#] | Randomization | Treatment 1 | Treatment 2 | Treatment 3 | Follow-up |
| | Time | Days/Weeks | Day 0 | Day 0 | Day 7 (≥ 7 days)* | Day 14 (≥ 7 days)* | Day 21 (≥ 7 days)* |
| Informed Consent | | X | | | | | |
| Inclusion / Exclusion Criteria | | X | X | X | X | X | X |
| Medical History | | X | X | X | X | X | X |
| Concomitant/change in medication | | X | X | X | X | X | X |
| Physical Examination | | X | | X | X | X | X |
| Body weight and height | | X | | X | X | X | X |
| Vital Signs (BP, PR) | | X | | X | X | X | X |
| 12-lead ECG | | X | | X | X | X | X |
| Laboratory Tests | | X | | X | X | X | X |
| Pregnancy Test | | X | | X | X | X | X |
| Study Drug Dispensing / Return | | | | X | X | X | X |
| Adverse Events | | | | X | X | X | X |

*A minimum of 7 days as a washout period was planned.
# The screening visit included a physical examination, laboratory evaluation for hematology, clinical chemistry, virology and coagulation tests as well as ECG assessment and non-invasive arterial blood pressure measurement.

Inclusion criteria:

- Healthy volunteers $\geq$18 years of age, <65 years of age
- Ability to comprehend the nature of the study and its associated risks and discomforts
- Willingness to comply with the studies safety requirements (e.g. no full contact sport after intake of anticoagulants)
- Willingness to perform reliable contraception for the duration of the study
- Normal findings in the medical history and the physical examination, unless considered irrelevant by the investigator
- Normal liver or renal function during the screening visit

Exclusion criteria:

- Intake of any drugs that may interfere with the aims of the study (i.e. CYP inducer or inhibitors, other laxatives or anti-diarrheal drugs)
- Acute illness
- Pregnancy or breastfeeding
- Allergy or intolerances to any of the used products or product constituents
- History of bleeding or coagulation disorder
- Intake of direct oral anticoagulants or any contraindication against intake of 150mg dabigatran
- Planned intervention or surgery

Sample size calculation:

The sample size calculation was performed using G-Power Software (G*Power 3.1). We assumed that the primary endpoint, the AUC of dabigatran is normally distributed. Separate repeated measures ANOVAs were conducted for each primary endpoint, with treatment period as the within-subjects factor.
A 25% change in AUC was considered a mild to moderate interaction according to the classification of the US Food and Drug Administration.(Huang et al., 2007) A variability of dabigatran plasma levels had been noted in previous studies. However, based on the low oral bioavailability of only 6%, we

assumed a greater impact of laxative treatment on the AUC of dabigatran. In healthy volunteers the mean AUC was 904± 397 ng*h/mL. (Stangier et al., 2007) Assuming a 40% decrease in the AUC (904 vs. 542 and SD of 397ng*h/ml) 16 healthy volunteers would have been sufficient to show a statistically significant difference with an alpha error of 5% and a power of 80%.
In order to increase the accuracy of our results and to account for potential drop outs 24 healthy volunteers were included in this study.

Randomization:
Healthy volunteers were randomized into six treatment groups. In the first study period, participants assigned to treatments 1-3 received a placebo with the study drug (dabigatran), while in the second study period, these participants received lactulose with the study drug. Participants in treatments 4-6 received lactulose with the study drug during the first period, followed by a placebo and the study drug in the second period.
Participants in Treatments 1 and 4 followed a 4-hour time-gap regimen (delayed lactulose) between the laxative and study drug intake. Participants in Treatments 2 and 5 received macrogol with the study drug in a third study period, whereas those in Treatments 3 and 6 were assigned flaxseed along with the study drug in the third study period. (Supplementary figure 1)
Randomization was conducted using a GCP conform, web-based randomization software provided by the Medical University of Graz, without applying stratification. (https://www.meduniwien.ac.at/randomizer/; Institute for Medical Informatics, Statistics and Documentation, Medical University of Graz. )

**Supplementary table 2: Baseline Characteristics**

| | All (n=24) | Delayed lactulose (n=8) | Macrogol (n=8) | Flaxseed (n=8) |
|---|---|---|---|---|
| Sex | | | | |
| Female | 15 (63%) | 4 (50%) | 6 (75%) | 5 (63%) |
| Male | 9 (37%) | 4 (50%) | 2 (25%) | 3 (37%) |
| Age (years) | 36.1 ± 2.5 | 36.9 ± 4.8 | 33.8 ± 3 | 37.5 ± 4.7 |
| BMI (kg/m²) | 24 ± 0.7 | 24.7 ± 1.3 | 22.5 ± 0.8 | 24.9 ± 1.3 |

Values are presented as mean ± SEM (standard error of the mean) for continuous variables and n (%) for categorical variables.

**Supplementary table 3**: Adverse events:

| Severity | AE | Subject | Comment |
|---|---|---|---|
| Mild AEs | Haematoma chin | Subject 2 | Due to vasovagal syncope |
| | Haematoma elbow | Subject 2 | Due to vasovagal syncope |
| | Pain shoulder | Subject 2 | Due to vasovagal syncope |
| | Pain Knee | Subject 2 | Due to vasovagal syncope |
| | Dysmenorrhea | Subject 13 | |
| | Food poisoning | Subject 17 | |
| | Rhinitis | Subject 17 | |
| | Bronchitis | Subject 18 | |
| | Sinusitis | Subject 18 | |
| Moderate AEs | Vasovagal syncope | Subject 2 | |
| | Vertigo | Subject 5 | |
| | Pneumonia | Subject 12 | |
| Severe AEs | none | | |

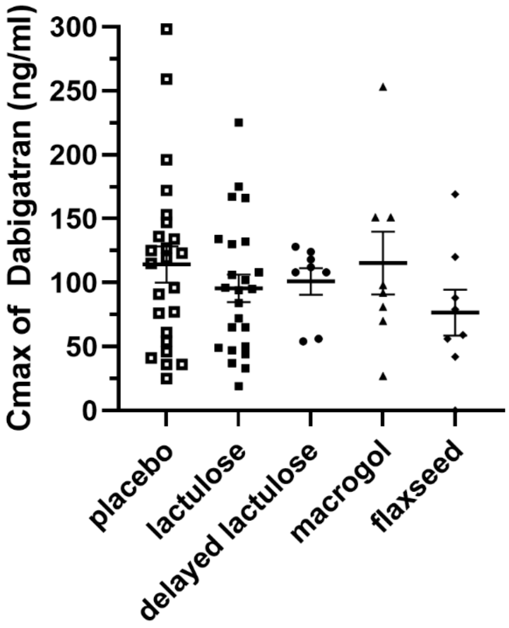


**Supplementary figure 1:** Maximum plasma concentration ($C_{max}$) of Dabigatran across different study groups. The data are presented as mean ± SEM, with individual measurements plotted to illustrate variability within each group.

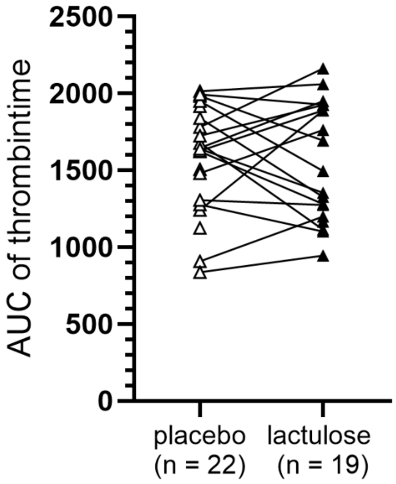


**Supplementary figure 2:** Individual changes in Thrombintime AUC between the placebo and lactulose groups. Each point represents the thrombin time AUC of a single participant over 24 hours, with lines connecting corresponding individual measurements across the two conditions.

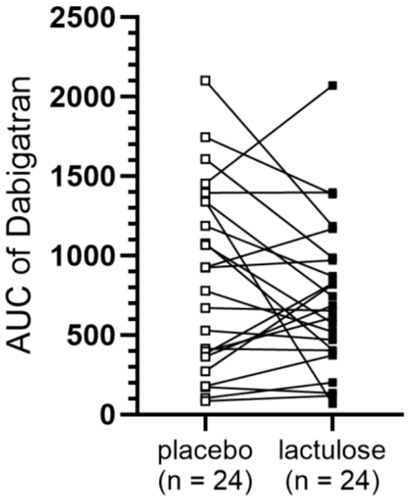


**Supplementary figure 3:** Individual changes in Dabigatran AUC between the placebo and lactulose groups. Each point represents the AUC of a single participant over 24 hours, with lines connecting corresponding individual measurements across the two conditions.

**References:**

Huang, S.-M., Temple, R., Throckmorton, D. C., and Lesko, L. J. (2007). Drug Interaction Studies: Study Design, Data Analysis, and Implications for Dosing and Labeling. *Clinical Pharmacology & Therapeutics* 81, 298–304. doi: 10.1038/sj.clpt.6100054

Stangier, J., Rathgen, K., Stähle, H., Gansser, D., and Roth, W. (2007). The pharmacokinetics, pharmacodynamics and tolerability of dabigatran etexilate, a new oral direct thrombin inhibitor, in healthy male subjects. *British Journal of Clinical Pharmacology* 64, 292–303. doi: 10.1111/j.1365-2125.2007.02899.x

**Supplementary table 4:** Laboratory Values for Safety Assessment Across Study Visits

| Laboratory Marker Mean (SEM) | Unit | Screening | Study-day_1_0h | Study-day_1_24h | Study-day_2_0h | Study-day_2_24h | Study-day_3_0h | Study-day_3_24h | EOS |
|---|---|---|---|---|---|---|---|---|---|
| Hemoglobin | g/dL | 13.2 (0.3) | 13.2 (0.3) | 13.0 (0.3) | 13.0 (0.3) | 12.8 (0.3) | 12.9 (0.3) | 12.8 (0.3) | 13.0 (0.3) |
| Hematocrit | % | 39.2 (0.8) | 39.1 (0.8) | 38.5 (0.7) | 38.5 (0.7) | 37.9 (0.7) | 38.1 (0.8) | 38.0 (0.8) | 38.5 (0.9) |
| Platelets | $10^9$/L | 249.3 (9.4) | 246.1 (11.5) | 246.8 (10.4) | 254.6 (12.6) | 261.2 (13.7) | 256.1 (12.6) | 256.4 (12.2) | 254.8 (11.7) |
| Leukocytes (WBC count) | $10^9$/L | 6.0 (0.4) | 5.7 (0.3) | 5.5 (0.3) | 5.7 (0.4) | 5.7 (0.3) | 5.9 (0.3) | 6.0 (0.3) | 5.7 (0.3) |
| Prothrombin Time (PT) | seconds | 84.4 (3.1) | 85.4 (3.1) | 80.6 (4.6) | 87.0 (3.4) | 88.0 (3.6) | 89.9 (3.5) | 86.6 (3.0) | 84.9 (3.5) |
| International Normalized Ratio (INR) | | 1.1 (0.0) | 1.1 (0.0) | 1.0 (0.1) | 1.1 (0.0) | 1.1 (0.0) | 1.1 (0.0) | 1.1 (0.0) | 1.1 (0.0) |
| Activated Partial Thromboplastin Time (aPTT) | seconds | 34.0 (0.5) | 35.1 (0.7) | 34.0 (1.7) | 34.8 (0.7) | 34.9 (0.5) | 34.1 (0.7) | 34.4 (0.5) | 33.6 (0.6) |
| Thrombin Time (TT) | seconds | | 19.8 (2.2) | 18.6 (3.0) | 20.9 (1.6) | 22.4 (2.3) | 18.8 (1.6) | 18.6 (3.1) | |
| Fibrinogen | mg/dL | 260.5 (7.2) | 259.2 (7.8) | 247.2 (13.6) | 267.3 (9.7) | 258.8 (9.0) | 262.4 (8.9) | 273.9 (8.8) | 263.5 (9.4) |
| Creatinine | mg/dL | 0.9 (0.0) | 0.9 (0.0) | 0.9 (0.0) | 0.9 (0.0) | 0.9 (0.0) | 0.9 (0.0) | 0.9 (0.0) | 0.9 (0.0) |
| Urea (BUN) | mg/dL | 13.7 (0.9) | 12.7 (1.0) | 12.7 (1.0) | 12.9 (0.8) | 13.6 (0.9) | 13.8 (0.9) | 13.0 (1.0) | 13.0 (1.0) |
| Aspartate Aminotransferase (AST, GOT) | U/L | 22.8 (1.3) | 23.7 (2.2) | 22.6 (2.1) | 21.7 (1.5) | 21.6 (1.2) | 22.2 (1.4) | 22.0 (1.9) | 22.3 (1.5) |
| Alanine Aminotransferase (ALT, GPT) | U/L | 22.0 (2.3) | 24.3 (3.5) | 23.0 (3.0) | 21.2 (1.9) | 21.6 (2.2) | 21.9 (2.1) | 22.0 (2.4) | 23.0 (2.8) |
| Total Bilirubin | mg/dL | 0.6 (0.1) | 0.6 (0.1) | 0.5 (0.1) | 0.5 (0.1) | 0.5 (0.1) | 0.5 (0.1) | 0.5 (0.1) | 0.5 (0.1) |
| C-Reactive Protein (CRP) | mg/dL | 0.1 (0.0) | 0.1 (0.0) | 0.1 (0.0) | 0.2 (0.1) | 0.1 (0.0) | 0.1 (0.0) | 0.1 (0.0) | 0.1 (0.0) |
| Sodium (Na) | mmol/L | 138.8 (0.5) | 139.3 (0.5) | 139.7 (0.4) | 139.6 (0.4) | 139.7 (0.4) | 139.8 (0.6) | 140.2 (0.4) | 139.6 (0.4) |
| Potassium (K) | mmol/L | 4.2 (0.1) | 4.3 (0.1) | 4.3 (0.1) | 4.3 (0.1) | 4.3 (0.1) | 4.3 (0.1) | 4.4 (0.1) | 4.4 (0.1) |
| Chloride (Cl) | mmol/L | 103.0 (0.5) | 103.7 (0.5) | 104.1 (0.5) | 104.0 (0.4) | 104.4 (0.5) | 104.0 (0.6) | 103.9 (0.5) | 104.1 (0.5) |
| Lactate Dehydrogenase (LDH) | U/L | 156.9 (6.6) | 154.6 (6.1) | 154.0 (6.1) | 156.1 (6.4) | 151.9 (5.6) | 155.4 (5.8) | 142.3 (8.2) | 151.4 (5.9) |

Mean values with standard error of the mean (SEM) in parentheses of various laboratory markers measured at study day 1 – 3 and the day after (24h). EOS: end of study.
